# Innovations in craniosynostosis surgery

**DOI:** 10.3171/2021.1.FOCVID20113

**Published:** 2021-04-01

**Authors:** Craig Birgfeld, Federico Di Rocco, Cormac O. Maher, Mark R. Proctor, Matthew D. Smyth

**Affiliations:** 1 Department of Plastic Surgery, Seattle Children's Hospital, University of Washington, Seattle, Washington;; 2 Department of Pediatric Neurosurgery, Université Claude Bernard, Lyon, France;; 3 Department of Neurosurgery, University of Michigan, Ann Arbor, Michigan;; 4 Department of Neurosurgery, Boston Children's Hospital and Harvard Medical School, Boston, Massachusetts; and; 5 Department of Neurosurgery, St. Louis Children's Hospital, Washington University, St. Louis, Missouri

Our understanding of craniosynostosis has evolved substantially over the past several decades. Once a condition treated almost exclusively in the realm of neurosurgeons, craniosynostosis is now the focus of a very robust community of craniofacial surgeons and neurosurgeons working together for the care of these complex patients. Indeed, perhaps one of the greatest changes is the understanding that these conditions can be far more complicated than previously understood and have lifelong implications for the child with regard to the aesthetic and neurocognitive outcomes. It is now widely accepted that children with these conditions benefit from care at craniofacial centers that offer expertise in all aspects of their management.

In addition to improved understanding of the condition, there have been many innovations in technology that have led to treatment evolutions over the past several decades. Some of these innovations include minimally invasive surgery employing endoscopes as well as postoperative adjuvant therapy such as placement of springs or the use of external orthoses to guide cranial growth. From a teaching and training perspective, the use of simulation also offers substantial potential benefits, especially for rare and complex conditions for which a 3D model can allow the surgeon to plan and practice the operation before an incision is made. Finally, other technologies such as external distractors and new devices such as piezoelectric surgery for bone cutting are also changing the field.

In this exciting video journal dedicated to craniosynostosis, the authors evaluate some of the basic techniques and then describe the use of these new and innovative techniques for the treatment of single-suture and multisuture syndromic synostosis. The reader should be able to appreciate many of the nuances in the treatment of these disease processes and see how surgeons are dealing with the conditions using the new innovations available in the modern era.

## Acknowledgments

We would like to acknowledge the passing of a master in the field of craniofacial surgery, Dr. James Goodrich, whom we lost this past year. He was a pioneer in his techniques and known for his immense dedication to the field and to the children with these conditions. It is a sad loss for our community.

